# Supplementary material for: The causal relationship between the human gut microbiota and pyogenic arthritis: a Mendelian randomization study
Source: Front Cell Infect Microbiol. 2024 Nov 26;14:1452480. doi: 10.3389/fcimb.2024.1452480 (PMC11629706; doi:10.3389/fcimb.2024.1452480)
Supplement: Supplementary file 1 [file DataSheet1.zip › mendelian test/ebi-a-GCST90027519.csv_forest.pdf]

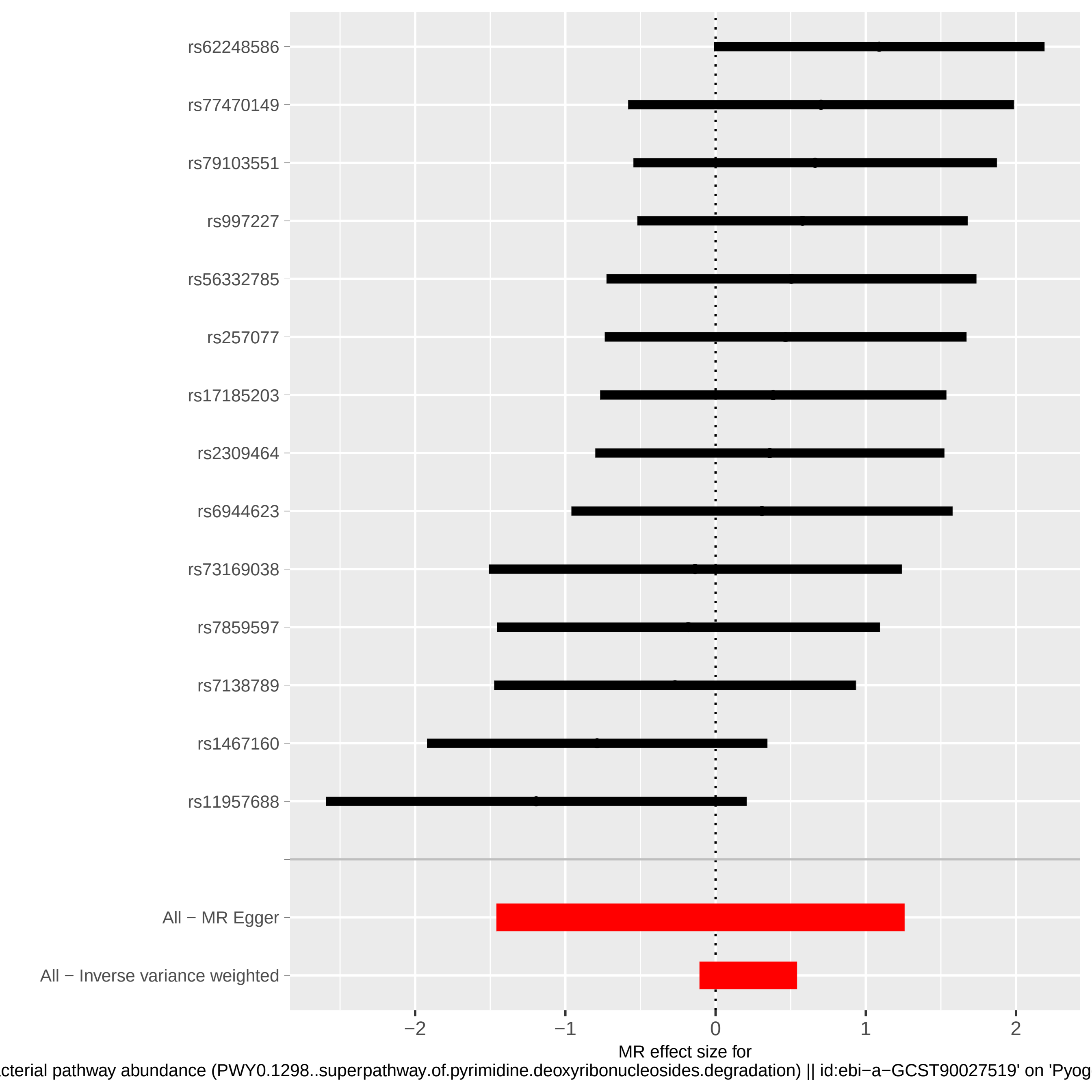
rs62248586
rs77470149
rs79103551
rs997227
rs56332785
rs257077
rs17185203
rs2309464
rs6944623
rs73169038
rs7859597
rs7138789
rs1467160
rs11957688
All − MR Egger
All − Inverse variance weighted
−2
−1
0
1
2
MR effect size for
cterial pathway abundance (PWY0.1298..superpathway.of.pyrimidine.deoxyribonucleosides.degradation) || id:ebi−a−GCST90027519' on 'Pyog
